# Phase Behavior and Proton Conductivity in Crown Ether-Based Supramolecular Sodium Hydrogen Sulfate Complexes

**DOI:** 10.1021/acs.cgd.5c01467

**Published:** 2025-12-15

**Authors:** Andrea Vitale, Samet Ocak, Antunes Staffolani, Francesca Soavi, Simone Bordignon, Michele R. Chierotti, Simone d’Agostino

**Affiliations:** a Department of Chemistry “Giacomo Ciamician”, 9296 The University of Bologna, Via P. Gobetti 85, 40129, Bologna (BO) 40126, Italy; b Center for the Environment, Energy, and Sea−Interdepartmental Centre for Industrial Research in Renewable Resources, Environment, Sea and Energy (CIRI-FRAME), Alma Mater Studiorum University of Bologna, Viale Ciro Menotti, 48, Marina di Ravenna (RA) 48122, Italy; c National Reference Center for Electrochemical Energy Storage (GISEL)—INSTM, Via G. Giusti 9, Firenze 50121, Italy; d Dipartimento di Chimica and NIS Centre, University of Torino, Via P. Giuria, 7, Torino 10125, Italy

## Abstract

This study deals with the preparation and solid-state characterization, as well as structural and phase transition features of supramolecular complexes composed of sodium hydrogen sulfate (NaHSO_4_) and two crown ether ligands, namely, 15-crown-5 and benzo-15-crown-5. Single crystals for each compound were grown, and their structures were elucidated via single-crystal X-ray diffraction (XRD) analysis, which highlighted the following compositions: [15-crown-5·Na]­HSO_4_ (**1**) and [benzo-15-crown-5·Na]­HSO_4_ (**2**). Microcalorimetric analyses, hot-stage microscopy, and variable-temperature powder X-ray diffraction were employed to analyze thermal stability and phase transition behaviors. Variable-temperature ^1^H *T*_1_ solid-state NMR measurements were also used to monitor proton dynamics and to determine activation energies associated with motion across phase transitions. Formation of supramolecular complexes is crucial for inducing solid–solid transitions, leading to superprotonic phases, namely, crystalline solids exhibiting an enhanced ability to conduct protons, as demonstrated through electrochemical impedance spectroscopy measurement.

## Graphical abstract

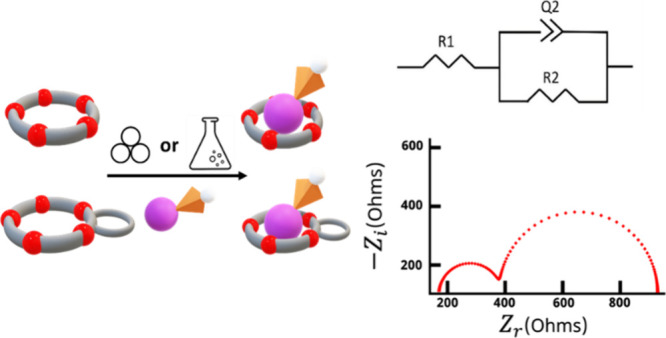

## Introduction

The design and development of solid electrolytes, materials known for their ion conduction properties, have garnered significant attention in recent decades. These materials are crucial for applications in various electrochemical devices, including molecular sensors, supercapacitors, batteries, and fuel cells.^1–5^ Several key advantages motivate the development and adoption of solid electrolytes over liquid counterparts, including nonflammability, enhanced chemical and thermal stability, and design flexibility.^6–8^

Among solid electrolytes, proton conductors form a specific subclass where hydrogen ions act as charge carriers. Nafion, an organic fluorinated polymer with sulfonic acid groups, is a notable example, exhibiting conductivity in the range of 10^–1^ to 10^–5^ S·cm^–1^.^9–11^

However, its conductivity is significantly influenced by factors such as hydration state, thermal history, and processing conditions.^12,13^ Consequently, current research focuses on discovering new materials that can conduct protons effectively in dry environments or at low temperature.^14–16^

Alternatives based on other polymers, Metal Organic Frameworks (MOFs), Covalent Organic Frameworks (COFs), metal oxides, have been proposed so far and have shown promising features for proton conductivity.^11,17–23^ However, achieving high proton conductivity in most of these materials still necessitates moisture or hydrated conditions, posing a significant drawback for sustained efficiency over time and at elevated temperatures. Further alternatives are represented by Ionic Plastic Crystals (IPCs) and dynamic crystals.^24^ In these materials, chemical species retain a fixed position within a lattice structure but can rotate or reorient freely, much like the molecules in a liquid upon the application of external stimuli such as temperature or pressure.^25–28^ This unusual combination of solid and liquid properties endows such a class of crystalline materials with unique physical characteristics such as high ionic conductivity, and mechanical properties.^29^ Another alternative is represented by the use of solid acids, i.e., compounds with general formula MHAO_4_ and MH_2_BO_4_ (where M = alkali cation, A = S or Se; and B = P or As).^30,31^ Proton conduction in these crystalline materials occurs through a structural diffusion mechanism, known as the Grotthuss mechanism,^32^ and is due to the insurgence of dynamically disordered hydrogen-bond networks associated with the onset of reversible, first-order solid–solid transitions.^33,34^

Unlike CsHSO_4_, which features a series of solid–solid transitions leading to a disordered superprotonic phase,^35–38^ other hydrogen sulfates of alkali metals like KHSO_4_ and RbHSO_4_ do not show such behavior.^39^ For NaHSO_4_ an irreversible phase transition, mediated by heat and water, was reported between two ordered anhydrous modifications that do not possess superprotonic phase features.^40^

In previous studies, we and others have shown how supramolecular complexation of solid-acids with formula MHAO_4_ (where M = K, Rb, or Cs; and A = S or Se) with the 18-crown-6 ether ligand enables enantiotropic solid–solid transitions associated with the onset of dynamical processes affecting both the crown ether ligand and the anion, leading to superprotonic phases.^41–43^ Additionally, we have also achieved fine-tuning for the transition temperature by anion replacement and solid solution formation.^44–46^

In our ongoing quest to discover novel crystalline materials capable of exhibiting solid–solid transitions and leading to superprotonic phases, we have synthesized, applying crystal engineering principles,^47–49^ a series of supramolecular complexes consisting of sodium hydrogen sulfate (NaHSO_4_) as the solid acid and 15-crown-5 and benzo-15-crown-5 as the ligands (see Scheme 1a).

**Scheme 1:**
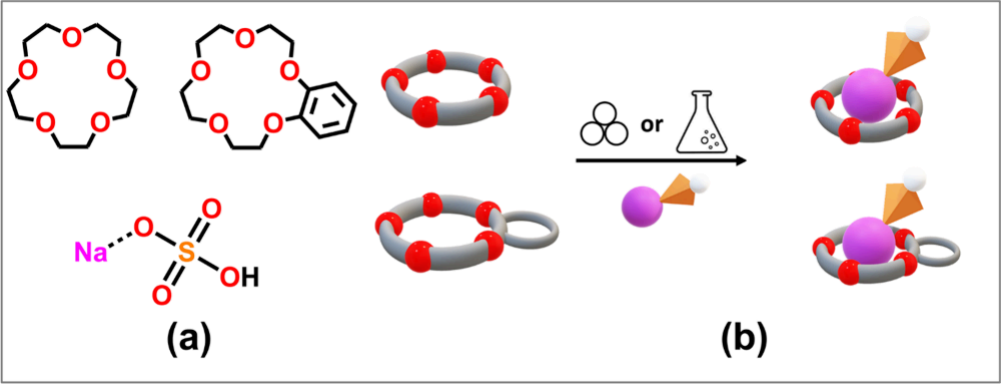
(a) Molecular Structures of the Crown Ethers Chosen As Components for the Preparation of Supramolecular Complexes with Sodium Hydrogen Sulfate (NaHSO_4_); (b) Solid-State Products of Mechanochemical and Solution Reactions of 15-Crown-5 and Benzo-15-crown-5 with NaHSO_4_

We deliberately selected these crown ethers with varying shapes to investigate how different macrocyclic ligands influence the packing features and solid-state phase transition in terms of temperature and type of ligand, as well as the thermal stability of the resulting materials.

Crown ethers exhibit a pronounced affinity for the alkali metal cations whose sizes align with the dimensions of their binding cavities, defined by the surrounding O atoms.^50,51^ As a matter of fact, both 15-crown-5 and benzo-15-crown-5 exhibit a strong preference for binding Na^+^ within their cavities, whereas the coordination is completed by water molecules or anions interacting directly with the metal ion.

However, due to its protruding and bulky nature, the benzo moiety is expected to be more easily “locked in place” by the surrounding molecules, thus providing, a marked effect on the phase transition and, eventually, on the proton transport via Grotthuss mechanism.^32^

With this in mind, we reacted solid acid NaHSO_4_ with each of the crown ethers (Scheme 1b) to form supramolecular complexes. Polycrystalline samples have been obtained through conventional slow evaporation or mechanochemically. Subsequently, we have grown single-crystal samples and employed X-ray Diffraction (XRD) data to elucidate their structures, discerning the distinctive structural variations induced by diverse macrocyclic ligands.

Two novel complexes, namely [15-crown-5·Na]­HSO_4_ (**1**) and [benzo-15-crown-5·Na]­HSO_4_ (**2**), have been obtained and characterized. Microcalorimetric analyses, such as Thermogravimetric Analysis (TGA) and Differential Scanning Calorimetry (DSC), were used to study their phase transition behaviors. Variable Temperature Powder XRD (VT PXRD) was essential to further analyze and confirm the microcalorimetric results. In addition, proton dynamics and the corresponding activation energies were also explored using variable-temperature ^1^H *T*_1_ solid-state NMR (SSNMR) measurements. Finally, Electrochemical Impedance Spectroscopy (EIS) was successfully applied in the anhydrous environment of a dry room, to prove and study the proton conduction features associated with temperature variations and the solid–solid phase transition.

## Experimental Section

### Synthesis

All reactants and reagents were purchased from Sigma-Aldrich and used without further purification. Reagent-grade solvents and bidistilled water were used. Crystalline reactants were checked by powder XRD analysis prior to use (see below). The supramolecular complexes have been prepared with similar procedures, according to previous results obtained and from literature.^52^

In a typical mechanochemical reaction, the crown ether and NaHSO_4_·H_2_O were mixed in the 1:1 stoichiometric ratio (refer to Table 1 for specific amounts) and ground together for 10 min using a pestle and agate mortar. Alternatively, the supramolecular complexes can be synthesized through conventional solution methods using either ca. 10 mL of water (H_2_O) or methanol (MeOH) as the solvent. In some syntheses a slight excess of crown ether was added to push the reaction to completion. In this case, the excess crown ether in the resulting solids was removed by washing (5 × 2 mL) with diisopropyl ether (DIPE). Single-crystal specimens suitable for XRD analysis were obtained by slow evaporation of water or methanol solutions at room temperature, resulting in plate-like crystals, subsequently isolated and washed with DIPE (5 × 2 mL) prior to be identified as [15-crown-5·Na]­HSO_4_ (**1**) and [benzo-15-crown-5·Na]­HSO_4_ (**2**). Elemental analysis (%) calculated for **1**: C, 35.29; H, 6.22; Na, 6.76; O, 42.31; S, 9.42. Found: C, 34.23; H, 5.90; S, 8.79. Elemental analysis (%) calculated for **2**: C, 43.30; H, 5.45; Na, 5.92; O, 37.08; S, 8.26. Found: C, 42.9; H, 6.03; S, 8.37.

**Table 1:** Amounts of Reagents Used in the Solution and Mechanochemical Synthesis of the Supramolecular Complexes: [15-Crown-5·Na]­HSO_4_ (**1**) and [Benzo-15-crown-5·Na]­HSO_4_ (**2**)

	NaHSO_4_ (mg/mmol)	15-crown-5 (mL/mmol)	benzo-15-crown5 (mg/mmol)
**1**	125.0/0.908	0.18/0.908	
**2**	200.0/0.745		102.0/0.745

### X-ray Diffraction (XRD)

Single-crystal XRD data for [15-crown-5·Na]­HSO_4_ (**1**) at Room Temperature (RT) and for [benzo-15-crown-5·Na]­HSO_4_ (**2**) at RT and Low Temperature (LT) (100 K/–170 °C) were collected on an Oxford X’Calibur S CCD diffractometer equipped with a graphite monochromator (Mo Kα radiation, λ = 0.71073 Å) and an Oxford CryoStream800 cryostat. In each case, crystals showed twinning, and the reflection data were integrated with the default configuration for twinned crystals of the CrysAlisPro Software. The structural solution and refinement were performed using the HKLF4 file containing non-overlapped reflections.

All the structures were solved with SHELXT by intrinsic phasing^53^ and refined on F^2^ with SHELXL^54^ implemented in the Olex2 software^55^ by full-matrix least-squares refinement. H_OH_ atoms were either directly located or, when not possible, added in calculated positions; H_CH_ atoms for all compounds were added in calculated positions and refined by riding on their respective carbon atoms. All non-hydrogen atoms were anisotropically refined and the rigid-body RIGU restraints^56^ applied. Data collection and refinement details are listed in Table S1. The Mercury software^57^ was used for molecular graphics and calculation of intermolecular interactions.

Phase identification and variable-temperature powder X-ray diffraction measurements were carried out using a PANalytical X’Pert PRO automated diffractometer, which was equipped with an X’Celerator detector in Bragg–Brentano geometry. Cu Kα radiation (λ = 1.5418 Å) was employed without a monochromator, within a 2θ range of 3° to 40° (continuous scan mode, step size 0.0167°, counting time 1.685 s, soller slit 0.04 rad, antiscatter slit 1/2, divergence slit 1/4, 40 mA, 40 kV). An Anton-Paar TTK 450 + LNC was also used. Mercury software was employed to calculate the powder XRD patterns based on the single-crystal data collected in this study. In all cases, the identity between polycrystalline samples and single crystals was always verified by comparing experimental and calculated powder diffraction patterns from this study or retrieved from CCDC^58^ or ICSD^59^ (See Figures S1–S3).

### Infrared Spectroscopy (ATR-FTIR)

Attenuated total reflectance Fourier transform IR (ATR-FTIR) spectra were obtained using a Bruker Alpha FT-IR spectrometer. ATR-FTIR spectra were run on polycrystalline samples of [15-crown-5·Na]­HSO_4_ (**1**) and [benzo-15-crown-5·Na]­HSO_4_ (**2**) and compared with the starting materials. See Figures S4 and S5.

### Thermogravimetric Analysis (TGA)

TGA analyses were performed with a PerkinElmer TGA 8000 instrument. Each sample, contained in a platinum crucible, was heated in a nitrogen flow (20 cm^3^·min^–1^) at a rate of 5 °C·min^–1^, up to decomposition. Samples weights were in the range 5–10 mg. See Figure S6.

### Differential Scanning Calorimetry (DSC)

Calorimetric measurements were performed with a PerkinElmer DSC-7 instrument equipped with a PII intracooler. Temperature and enthalpy calibrations were performed on high-purity standards (*n*-decane, benzene, and indium). Heating of the aluminum open pans containing the samples (3–5 mg) was carried out at 5 °C·min^–1^ in the temperature range 20–120 °C under N_2_ atmosphere. See Figures S7 and S8.

### Hot Stage and Cross-Polarized Optical Microscopy

Hot stage experiments were carried out using a Linkam TMS94 device connected to a Linkam LTS350 platinum plate and equipped with a NIKON DS F13 CCD camera, from an Olympus BX41 stereomicroscope.

### Variable-Temperature Solid-State NMR (VT SSNMR)

SSNMR experiments were run on a Jeol ECZR 600 instrument, operating at a frequency of 600.13 and 150.91 MHz for ^1^H and ^13^C, respectively and equipped with a 3.2 mm probe. A 3.2 mm zirconia rotor (o.d. = 60 μL) specific for high-temperature analyses was packed with an appropriate amount of **1** or **2**. The ^1^H MAS spectra for **1** were acquired with the DEPTH sequence (π/2−π–π) for the suppression of the probe background signal at different temperatures at a spinning speed of 12 kHz (^1^H 90° = 2.5 μs; 16 scans; optimized relaxation delays ranging from 2.8 to 11.1 s, depending on the temperature). ^1^H *T*_1_ values for **1** were measured at variable temperature for the ∼4.0 ppm signal relative to crown ether protons by means of ^1^H saturation recovery (1 scan, exponential τ values ranging 0.1–100 s). ^13^C CPMAS spectra were acquired at a spinning speed of 12 kHz, using a ramp cross-polarization pulse sequence with a 90° ^1^H pulse of 2.0 μs, contact time of 3.5 ms, optimized recycle delays between 2.8 and 11.1 s, number of scans in the range 16–40, depending on the sample. The ^1^H chemical shift scale was calibrated with adamantane (^1^H signal at 1.87 ppm with respect to the primary standard tetramethylsilane) as an external standard. Temperature calibration on this probe was performed by acquiring ^207^Pb MAS spectra on external standard Pb­(NO_3_)_2_. The complete list of set and corresponding sample temperatures is reported in Table S12.

### Electrochemical Impedance Spectroscopy (EIS)

The ionic conductivity was assessed by Electrochemical Impedance Spectroscopy (EIS) at different temperatures. A VSP-3a (Bio-Logic SAS, Seyssinet-Pariset, France) potentiostat/galvanostat/frequency analyzer was used for the acquisition of the Nyquist plots. The measurements were performed in two-electrodes configuration with stainless-steel blocking electrodes. The powders were pressed into pellets with Ø = 13 mm and each side of them was coated with an Ag paste (Elettro’340 Argento conductive paint) to ensure electronic contact with the cell current collectors. The pellets were then dried at 60 °C under vacuum (Büchi B-585, Cornaredo, Italy). The Nyquist plots were acquired by applying an AC perturbation Δ*E* = 50 mV at the open circuit potential, in the frequency range of 1 MHz < ν < 1 Hz and collecting 10 points per decade. EIS measurements were performed at different temperatures, starting from 60 °C up to 130 °C with 5 °C intervals by using a programmable static oven (Pol-Eko, Wodzisław Śląski, Poland). The cells were allowed to reach a thermal equilibration for 30 min before the next EIS acquisition. To avoid any effect of moisture, all measurements were performed in a dry room with a dew point of – 40 °C (il Disgelo, Torino, Italy).

The EIS spectra were fit by using an equivalent circuit model (ECM) consisting of a resistance (R) in parallel with a constant phase element (Q). The parallel branch is noted with (RQ) in Boukamp’s notation.^60^ Fitting was carried out by using the DearEIS software.^61^

## Results and Discussion

### Synthesis and Structural Description

We have focused our attention mainly on preparing the supramolecular complexes via mechanochemistry, i.e., manual grinding. To this end, the solid acid (NaHSO_4_·H_2_O) and liquid 15-crown-5 or solid benzo-15-crown-5 were ground together in the proper stoichiometric ratio in an agate mortar for 10 min (see Experimental section for details), and to assess complexation in the solid state the products were then split, one portion was directly analyzed with powder XRD, the second one was used to grow single-crystal specimens from solution for structural (vide infra) and powder analyses. Figure 1 compares the experimental powder XRD patterns for the starting materials and mechanochemical products, as well as the ones obtained via recrystallization from slow evaporation of methanol solution and calculated from the single-crystal structures. For complexes obtained by reacting NaHSO_4_·H_2_O with 15-crown-5 or benzo-15-crown-5 through different techniques, the powder XRD patterns are different compared to the ones from reactants and perfectly superimposable among them and to the ones calculated based on the respective crystal structures (*vide infra*). Infrared spectra were also recorded to confirm the complexation between the crown ethers and the solid acid NaHSO_4_ by monitoring the C–O stretching band,^62,63^ which moves as in our case, to lower frequencies upon complexation (see Figures S4 and SI5).^64,65^

**Figure 1:**
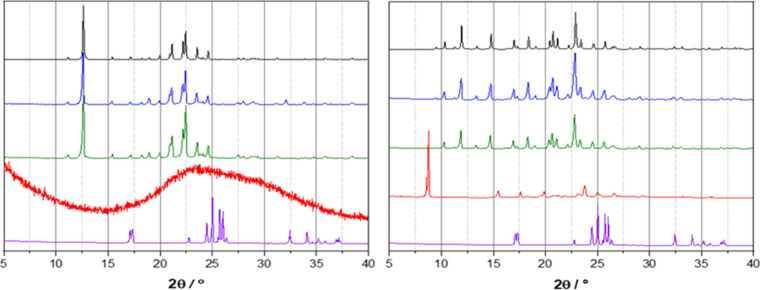
Comparison between powder XRD patterns recorded for the components NaHSO_4_·H_2_O (purple line) and crown ether ligands (red line): (left) 15-crown-5 and (right) benzo-15-crown-5, and the products obtained via different synthetic methods: manual grinding (green line) and slow evaporation (blue line), as well as with those calculated from single-crystal structures (black line).

Structural analyses at RT revealed a 1:1 composition for each complex, namely [15-crown-5·Na]­HSO_4_ and [benzo-15-crown-5·Na]­HSO_4_ for **1** and **2**, respectively, and showed that both compounds crystallized in the monoclinic *P*2_1_/*n* space group (see Table S1 for details). In each case, the metal cation Na^+^ interacts with the respective crown ether ligand, whereas the coordination sphere is completed by two additional O atoms from the hydrogen sulfate anion, as depicted in Figure 2 (see Table 2 for coordination distances). These anions also engage in charge-assisted hydrogen bonding interactions (see Table 2 for distances), forming supramolecular dimers, as shown in Figure 3. Close inspection reveals that the hydrogen sulfate anion within crystalline **2** at RT exhibits crystallographic disorder, which was modeled over two positions, and it can be “frozen out” on cooling down to −173 °C (100 K).

**Figure 2:**
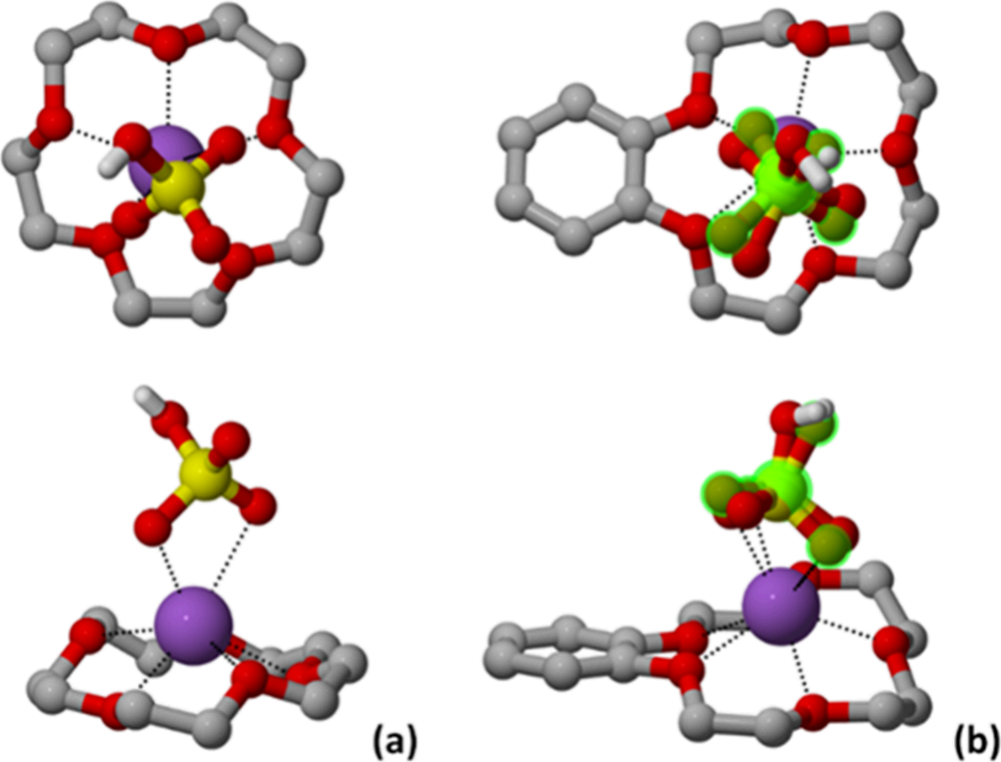
Top and side views showing the coordination geometry around the Na^+^ ions in crystalline **1** (left) and **2** (right). Disorder of the HSO_4_^–^ in **2** at RT highlighted in green, H_CH_ omitted for clarity.

**Figure 3:**
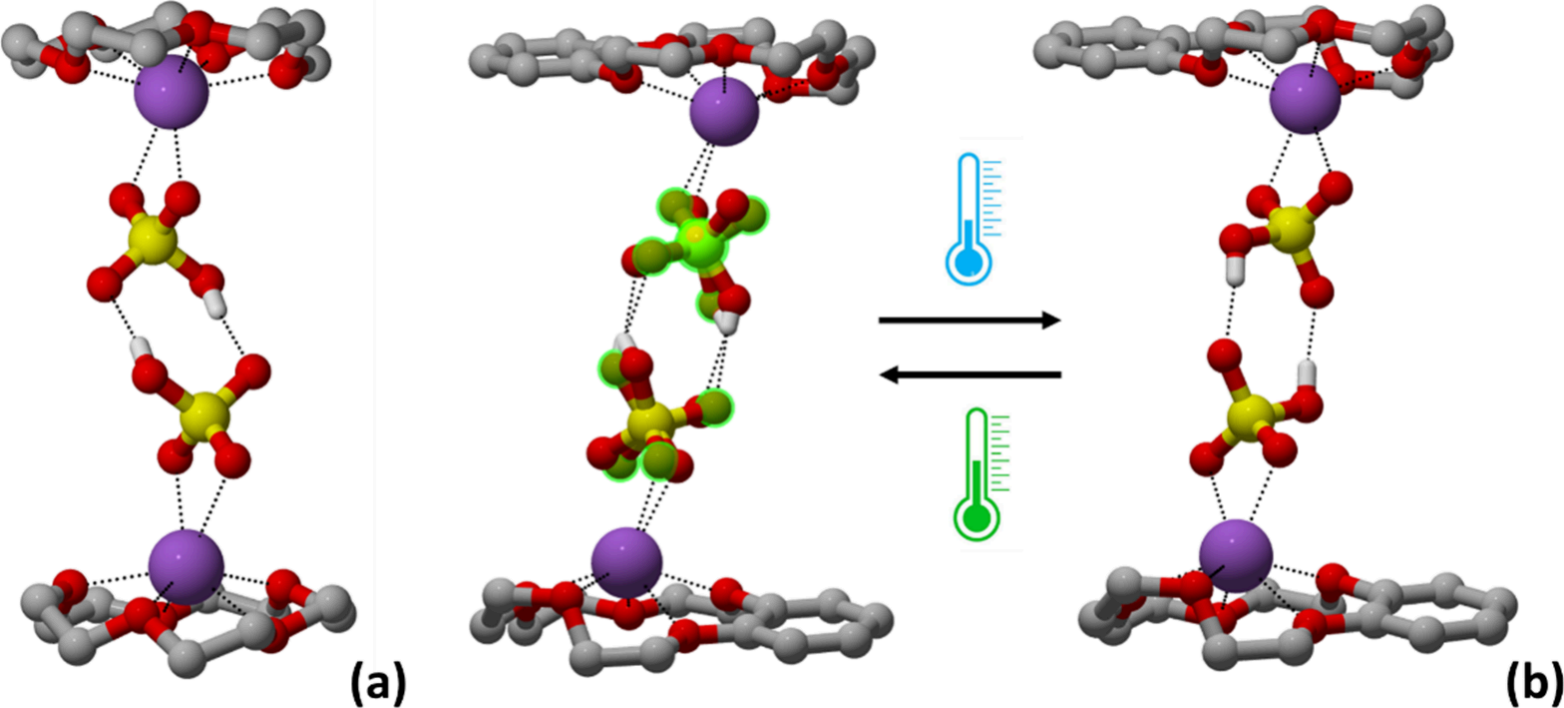
Hydrogen-bonded supramolecular dimers detected within crystalline: (a) **1** and (b) **2**, and the representation of the reversible disorder–order interconversion between the RT and −173 °C (100 K) structures. Disorder of the HSO_4_^–^ in **2** at RT highlighted in green, H_CH_ omitted for clarity.

**Table 2:** Coordination Distances and Hydrogen Bonding Interactions Detected Within Crystalline **1** and **2**

	coordination		hydrogen bonds
entry/temperature (K)	Na^+^···O_crown_ (Å)	Na^+^···O_anion_ (Å)	O_anion_··· O_anion_ (Å)
**1**/RT	2.392(5)–2.462(4)	2.403(4), 2.632(3)	2.640 (6)
**2**/RT	2.379(2)–2.486(3)	2.36(1)–2.74(1)	2.54(2)–2.64(2)
**2**/100 K	2.361(1)–2.495(2)	2.403(2), 2.592(2)	2.597(2)

Although both compounds share similar supramolecular dimeric assemblies, they exhibit different packing arrangements. In crystalline **1**, the dimers are packed to form channel-like sections (approximately 11 Å in diameter) running parallel to the crystallographic *a* and *b* axes, which host, quite accessible, hydrogen-bonded HSO_4_^–^ anionic pairs, as shown in Figure 4; whereas in crystalline **2**, the hydrogen-bonded HSO_4_^–^ anionic pairs are still hosted within section channels running parallel to the *c*-axis, but being surrounded by multiple [benzo-15-crown-5·Na]^+^ cationic units, they results more hindered (Figure 4).

**Figure 4:**
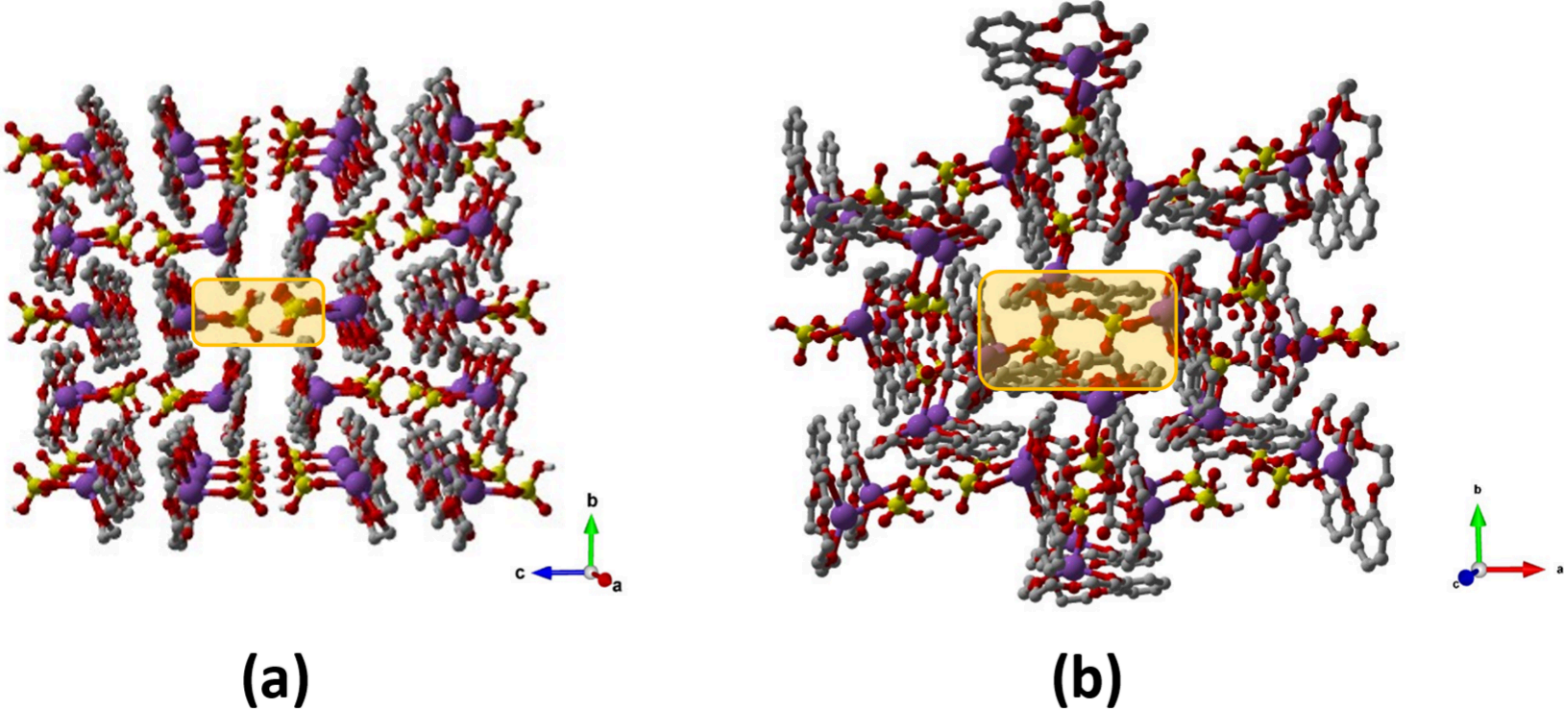
(a) Crystal packing of compound **1** viewed down the *a*-axis and (b) crystal packing of compound **2** viewed along the *c*-axis. H_CH_ atoms and disorder of the HSO_4_^–^ anions in compound **2** are omitted for clarity.

### Thermal Behavior and Phase Transitions

The thermal behavior of the two complexes [15-crown-5·Na]­HSO_4_ (**1**) and [benzo-15-crown-5·Na]­HSO_4_ (**2**) has been investigated to assess their thermal stability and identify any phase transition. These phase transitions, as noted in the introduction, could be closely linked to improved proton conduction within this class of compounds. TGA on polycrystalline **1** and **2** do not show any weight loss until 120 and 150 °C, respectively, indicating a good thermal stability. Thermograms for all compounds are shown in Figure S6.

DSC was employed to investigate any possible phase transition. Complex **1** shows an endothermic peak at 100.8 °C (Δ*H* = 2.6 kJ/mol) on heating, and an exothermic one at 87.8 °C (Δ*H* = −2.6 kJ/mol) on cooling mode, indicating thus a reversible transition (Figure S7). On the other hand, complex **2** exhibits no phase transition on heating (Figure S8).

Structural transformations were also monitored through VT PXRD. Measurements on a polycrystalline sample of complex **1** align well with the DSC analysis. Upon heating the material above the transition temperature, clear and distinct changes in the powder patterns are observed, indicating the formation of the **1**-HT phase (Figure S9). The process is fully reversible upon cooling. On the contrary, and as expected, for complex **2**, no changes occur in the powder XRD pattern collected upon heating (Figure S10).

To gain further insights into phase transitions and confirm the behavior of materials upon heating, Hot Stage Microscopy (HSM) has been utilized. Single crystals of **1** display, on heating, a marked change in birefringence (95–105 °C) according to the phase transition temperature found in DSC (Figure 5a). It is worth noting that the phase transition is accompanied by the formation of cracks leading to microcrystalline material in response to the tremendous pressure arising from the molecular rearrangement, like what happens during [2 + 2] solid-state reactions.^66,67^

**Figure 5:**
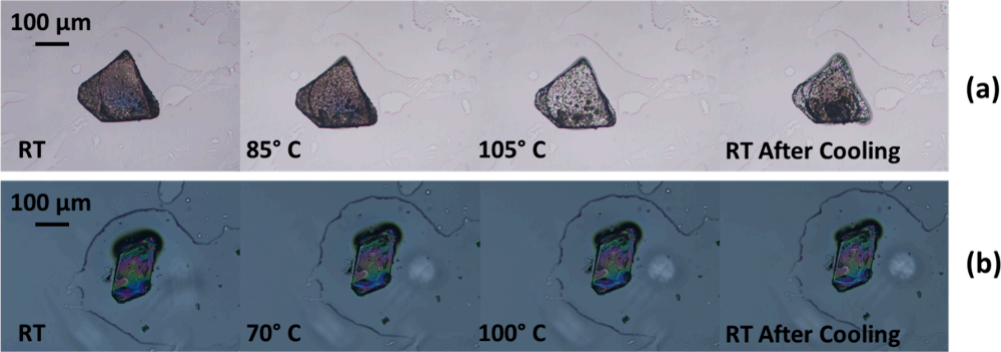
HSM images taken at increasing temperatures on single-crystal specimens of compounds (a) **1** and (b) **2**.

As expected, complex **2** shows consistency with DSC and VT PXRD results, since the single crystal, on heating, does not display any change in birefringence, crack formation, or crumbling (Figure 5b), thus indicating that no transition occurs.

### Solid-State NMR Spectroscopy

The ^13^C CPMAS spectra of **1** and **2** are reported in Figures S11 and S12 together with the list of chemical shifts and relative assignments shown in Table S3. The spectrum of **1**, as expected, is characterized by a single peak at 69.6 ppm ascribable to 15-crown-5, in agreement with the rapid rotation of the molecule around the C_5_ axis, even in the solid state. The spectrum of **2** is more complex, since the symmetry is lost and the rotation is hindered by the phenyl moiety: thus, it presents six signals for the CH_2_ groups and six resonances for the aromatic ring (see Table S3 for the chemical shifts).

Since in SSNMR the dynamics of a system are commonly investigated through key relaxation parameters, namely, *T*_1_, *T*_2_, and *T*_1ρ_, we focused on studying the ^1^H *T*_1_ values of relevant protons in the system. Indeed, as previously shown in the case of 18-crown-6 complexes,^41^ the ^1^H nucleus is particularly well-suited for studying the *T*_1_ relaxation of such proton-conducting materials. Here, since compound **2** did not display any phase transition, the ^1^H analysis was solely focused on compound **1.**

The ^1^H MAS SSNMR spectra of **1**, acquired at different temperatures, are shown in Figure S13. The main peak, at about 4 ppm, is ascribable to the 20 protons of the 15-crown-5 group; other two less intense signals appear around 11 and 7 ppm. To identify the resonance corresponding to the HSO_4_^–^ anion, a sample of compound **1** containing NaDSO_4_ in place of NaHSO_4_ was analyzed (Figure S14). This comparison allowed for the assignment of the lower-frequency resonance (δ = 7 ppm) to HSO_4_^–^, which was absent in the spectrum of the deuterated sample. However, further solid-state syntheses revealed the HSO_4_^–^ signal to resonate at different chemical shifts across batches, making it unreliable. Consequently, attention was directed to the crown ether signal instead. A significant feature of this peak is that it appears composed of two overlapping contributes, a sharper and a broader one, at similar chemical shifts. Thus, the ^1^H *T*_1_ values for both crown ether peaks were measured at variable temperatures by the saturation recovery pulse sequence to observe the presence of any difference in their associated dynamic behaviors. Figure 6 reports the ^1^H *T*_1_ relaxation times (in logarithmic scale) against the inverse of the absolute sample temperatures, for both components of the considered resonance. Set and sample temperatures in K and in °C are reported in Table S2.

**Figure 6:**
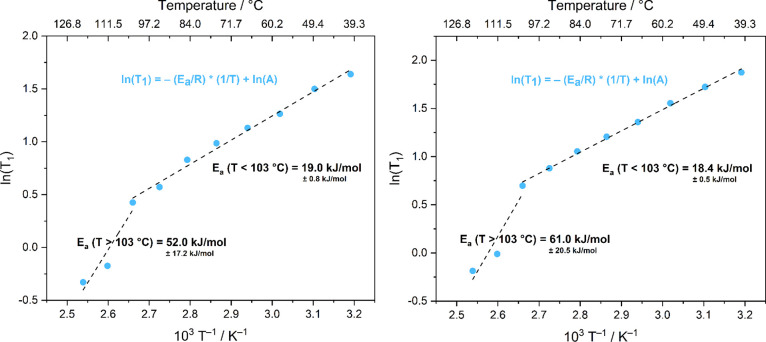
Sample temperature (K) dependence of ^1^H spin–lattice relaxation time (*T*_1_) for the crown ether signal peak (δ = 4.0 ppm) for compound **1**, in a logarithmic scale (with the respective general equation in blue). The top axis reports the corresponding temperatures in °C, for clarity. Left: sharp component; right: broad component. The *E*_a_ values corresponding to the slopes of the linear fits are reported.

In this kind of logarithmic plots, a change in the slope of the lines employed to fit the experimental data is associated to a variation in the occurring motional regimes, with the relative activation energy (*E*_a_) values directly derivable from said slopes. For compound **1**, a significant difference is observed before and after 103 °C for both signal components: at lower temperatures a dynamic process prevails, with *E*_a_ = 19.0 ± 0.8 kJ·mol^–1^ (sharp contribution) and *E*_a_ = 18.4 ± 0.5 kJ·mol^–1^ (broad contribution), while over 103 °C a different one ensues, characterized by *E*_a_ = 52.0 ± 17.2 kJ·mol^–1^ and *E*_a_ = 61.0 ± 20.5 kJ·mol^–1^, for the sharp and the broad components, respectively.

In both cases, the calculated activation energy values well agree with those reported in the literature for analogous systems.^68^

The data collected for the samples through the several employed techniques suggest that three processes, i.e., crown ether rotation, polymorphic transitions, and hydrogen sulfate proton mobility, occur independently of one another. This interpretation is supported by the observation that the transition temperatures and activation energy (*E*_a_) values obtained via SSNMR differ from those measured through DSC and EIS analyses (see below). Such differences are expected, as the three techniques inherently investigate different physical phenomena. Although these processes may, in some cases, influence one another, their distinct thermal behaviors suggest a lack of direct interdependence in this context.

### Electrochemical Impedance Spectroscopy (EIS) Study

Finally, to investigate the ion conducting features associated with the temperature and solid–solid phase transitions, EIS measurements were performed on polycrystalline samples **1** and **2** (see the Experimental section for details). To exclude any effect related to water adsorption from the atmosphere, EIS tests were run in a dry room. In Figure S15, the Nyquist plots of crystalline **1** and **2** are reported. The plots are all characterized by the presence of a semicircle with a diameter that decreases in magnitude as the temperature increases. This signature can be modeled referring to a (QR) equivalent circuit, where *R* represents the ionic resistance of the pellet and can be quantified with the semicircle diameter. Q is the double-layer capacitance of the cell which is set up by the arrangement of ions at the two-blocking electrode/pellet interfaces. In the case of **1**, a regular decrease of the semicircle diameter was observed with an increase of the temperature. Similarly, for compound **2** a proportional decrease of the impedance magnitude was observed with the increase of the working temperature. Fitting of the Nyquist plots provided the values of the pellet ionic resistance R.^60^ Then, the ionic conductivity σ (S·cm^–1^) was calculated^69^ according to the following equation:

$$\sigma =\frac{l}{RA}$$

in which *R* is the calculated resistance from the fitting procedure, *l* is the thickness (in cm) of the electrolyte pellet, and *A* is the geometrical area of the pellet (1.37 cm^2^). In all cases, the ionic conductivity increases almost linearly vs 1000 T^–1^ (expressed in K^–1^), suggesting a thermally activated process according to an Arrhenius behavior.^70,71^ Compound **1** shows ionic conductivity values of ca. 1.94·10^–7^ S·cm^–1^ (*T* = 60 °C) < σ < ca. 2.95·10^–6^·cm^–1^ (*T* = 110 °C). As in the Arrhenius conductivity plots (Figure 7a), no sudden improvements in ionic conductivity were observed apart from a small increase of the slope at *T* > 90 °C. By introducing benzene moieties in the ligand, i.e., in compound **2**, slightly lower ionic conductivity values were obtained.

**Figure 7:**
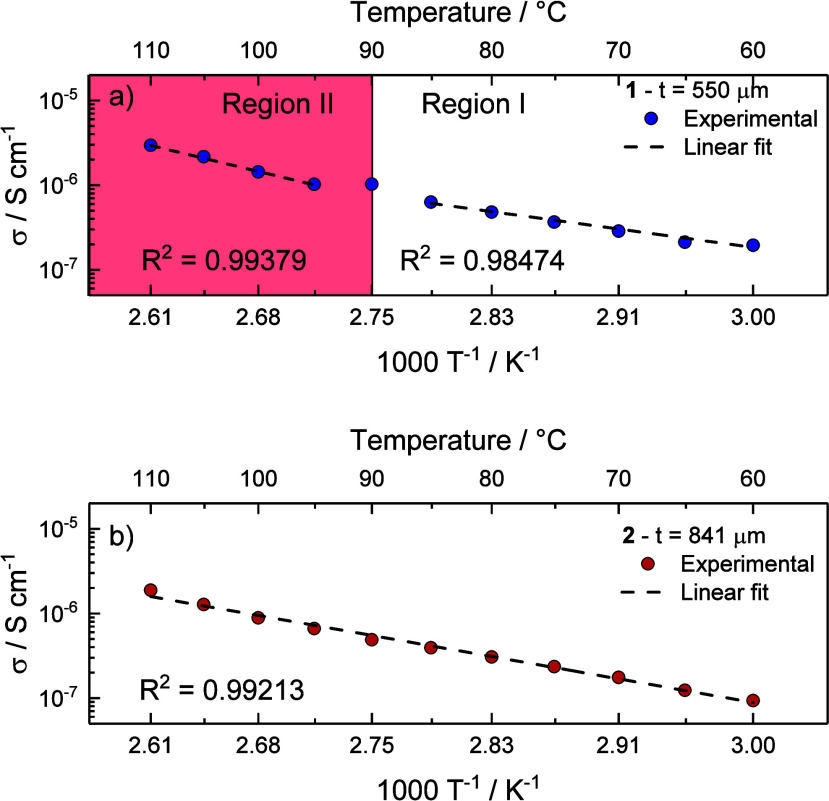
Arrhenius conductivity plots of (a) [15-crown-5·Na]­HSO_4_ (**1**) (pellet thickness *t* = 550 μm) and (b) [benzo-15-crown-5·Na]­HSO_4_ (**2**) (pellet thickness *t* = 841 μm).

Then, the activation energies of compounds **1** and **2** were calculated according to the following equation:^70^

$$\ln\sigma =\ln\sigma_{0}-\frac{E_{a}}{k_{b}T}$$

Where σ_0_ is the pre-exponential factor and graphically corresponds to the intercept of the linear fit, *E*_a_ is the activation energy, *k*_b_ is the Boltzmann’s constant (8.617·10^–5^ eV·K^–1^), and *T* is the temperature. The activation energy can be derived from the slope of the linear fit which is equal to *E*_a_/*k*_b_. The results obtained are summarized in Table 3. Two different linear regions were identified in the σ vs 1000 T^–1^ plot of compound **1**, i.e., Region I from 60 °C up to 85 °C and Region II from 95 °C up to 110 °C. In this case, activation energies of 48.56 kJ·mol^–1^ (0.504 eV) and 84.45 kJ·mol^–1^ (0.875 eV) were calculated for Region I and Region II, respectively. For compound **2** an *E*_a_ value of 61.18 kJ·mol^–1^ (0.634 eV). Considering the activation energy of compound **1** (Region I) at temperatures 60 °C < *T* < 85 °C, we can infer that the ion conduction is mainly given by proton ions with a Grotthuss mechanism (proton hopping mechanism), since it generally involves *E*_a_ < 0.5 eV.^72–74^ On the other hand, in Region II of compound **1** and in the whole temperature range of compound **2** activation energies accounts for 0.875 and 0.634, respectively. In general, *E*_a_ values >0.5 eV are referred to conduction processes involving the so-called “vehicle mechanism”, which involves the transport of larger ions (e.g., Na^+^) and thus a larger energy barrier. Thus, we can infer that the ion conduction at *T* > 90 °C in compound **1**, and compound **2** in the whole temperature range, is not solely given by protons but it may be given also by Na^+^ ions via vehicle mechanism.

**Table 3:** Ionic Conductivities at *T* = 60 °C and *T*= 110 °C and Activation Energies of Compounds **1** and **2**

	1		2	
	Region I	Region II		
σ/S·cm^–1^	1.94·10^–7^ (*T* = 60 °C)	2.95·10^–6^ (*T* = 110 °C)	9.34·10^–8^ (*T* = 60 °C)	1.88·10^–6^ (*T* = 110 °C)
*E*_**a**_ **/**kJ·mol^–1^	48.56	84.45	61.18	
*E*_a_/eV	0.504	0.875	0.634	
R^2^	0.98474	0.99379	0.99213	

## Conclusions

This study explored the synthesis, structural properties, thermal behavior, and proton conductivity of sodium hydrogen sulfate complexes with 15-crown-5 and benzo-15-crown-5 as ligands, deliberately selected to study how different shapes and conformations affect the properties of the resulting materials. Mechanochemical synthesis and slow evaporation were successfully applied to produce these supramolecular complexes, with distinct structural features. The obtained complexes [15-crown-5·Na]­HSO_4_ (**1**) and [benzo-15-crown-5·Na]­HSO_4_ (**2**) formed stable and anhydrous crystalline materials suitable for further thermal and electrochemical characterization.

Thermal analyses were used to investigate phase transitions in both complexes. Only compound **1** exhibited a reversible enantiotropic solid–solid phase transition, likely associated with the onset of dynamic disorder, which was confirmed by VT SSNMR spectroscopy measurements, whereas no phase transitions were detected for compound **2**. These differences could be due to the protruding and bulky nature of the benzo moiety, which prevents the insurgence of a phase transition promoted by the temperature increase. In addition, the analyses of ionic conductivity vs temperature, showed typical Arrhenius-type behavior for both compounds. In the case of compound **1**, two distinct regions were detected; at the lowest temperatures (*T* < 90 °C) the activation energy suggests that ion conduction is dominated by protons via a Grotthuss mechanism. At higher temperature (*T* > 90 °C), the insurgence of dynamic disorder affects ion transport, that becomes dominated by a vehicle mechanism, where, presumably, even Na^+^ ions are involved. On the other hand, when benzene moieties are introduced in the ligand, i.e., compound **2**, no transitions are present, and ion conduction is dominated by a vehicle mechanism in the whole tested temperature range (60 °C < *T* < 110 °C).

Overall, this study provides valuable insights for designing new materials with ion-conduction properties that could be useful in energy production and storage devices and emphasizes how ligand selection affects thermal stability, phase transitions, and conductivity, offering a comprehensive understanding of the structure–property relationships in crown ether-sodium hydrogen sulfate complexes. Work is ongoing to test new combinations of sodium salts and crown ethers.

## Supplementary Material



## Notes

Crystal data and refinement details, powder XRD patterns, ATR-FTIR spectra, thermal analyses, SSNMR spectra, and Nyquist Plots (PDF)

## e

Institute of Nanostructured Materials–Research National Council (ISMN-CNR), Via Pietro Gobetti 101, Bologna (BO) 40129, Italy (A.V.)
