# Supplementary material for: Phase Behavior and Proton Conductivity in Crown Ether-Based Supramolecular Sodium Hydrogen Sulfate Complexes
Source: Cryst Growth Des. 2025 Dec 15;26(1):589–98. doi: 10.1021/acs.cgd.5c01467 (PMC12787320; doi:10.1021/acs.cgd.5c01467)
Supplement: Supplementary file 1 [file cg5c01467_si_001.pdf]

Phase Behavior and Proton Conductivity in Crown Ether-Based Supramolecular Sodium Hydrogensulfate Complexes

Andrea Vitale,[a] Samet Ocak,[a,b] Antunes Staffolani,*[a,c] Francesca Soavi,[a,c] Simone Bordignon,[d] Michele R. Chierotti,*[d] and Simone d'Agostino*[a]

[a] The University of Bologna - Department of Chemistry "Giacomo Ciamician", Via F. Selmi 2, 40126, Bologna (BO), 40126, Italy.

[b] Current address: Institute for Microelectronics and Microsystems - Research National Council (IMM-CNR), Via Pietro Gobetti 101, 40129, Bologna (BO), Italy.

[c] Center for the Environment, Energy, and Sea - Interdepartmental Centre for Industrial Research in Renewable Resources, Environment, Sea and Energy (CIRI-FRAME), Alma Mater Studiorum University. of Bologna Viale Ciro Menotti 48, 48122 Marina di Ravenna (RA), Italy.

[d] Dipartimento di Chimica and NIS Centre, University of Torino, Via P. Giuria, 7, 10125 Torino, Italy.

Corresponding authors emails: simone.dagostino2@unibo.it; antunes.staffolani@unibo.it; michele.chierotti@unito.it

Table of contents	Page
Crystal data and refinement details	SI-2
Powder pattern comparison	SI-3
ATR-FTIR spectra	SI-5
Thermogravimetric analyses	SI-6
Differential scanning calorimetry traces	SI-7
Variable-temperature pxrd patterns	SI-8
Solid-state NMR spectroscopy	SI-9
Electrochemical Impedance Spectroscopy (Nyquist Plots)	SI-12

CRYSTAL DATA AND REFINEMENT DETAILS

Table SI1. Crystal data and refinement details for: [15-crown-5·Na]HSO_4 (**1**) at RT and [benzo-15-crown-5·Na]HSO_4 (**2**) at 100 K and RT.

	**1 (RT)**	**2 (100K)**	**2 (RT)**
**Formula**	$C_{10}H_{21}NaO_9S$	$C_{14}H_{21}NaO_9S$	$C_{14}H_{21}NaO_9S$
**FW (g/mol)**	340.33	388.36	388.36
**Cryst. Sys.**	monoclinic	monoclinic	monoclinic
**Space Group**	$P2_1/n$	$P2_1/n$	$P2_1/n$
**a/Å**	9.9522(7)	11.9217(4)	12.1259(2)
**b/Å**	10.1250(5)	12.0540(4)	12.2293(2)
**c/Å**	16.270(1)	12.1174(4)	12.1380(2)
**α/°**	90	90	90
**β/°**	103.385(7)	100.094(4)	99.854(2)
**γ/°**	90	90	90
**Volume/Å³**	1594.93(17)	1714.37(11)	1773.40(5)
Z	4	4	4
**ρ_{calc} g/cm³**	1.417	1.505	1.455
**μ/mm⁻¹**	0.268	0.260	0.251
**measd rflns**	6735	13167	26133
**indep rflns**	3608	4091	4488
**R_1**	0.0798	0.0456	0.0775
**wR_2**	0.2066	0.0930	0.2183

POWDER XRD PATTERN COMPARISON

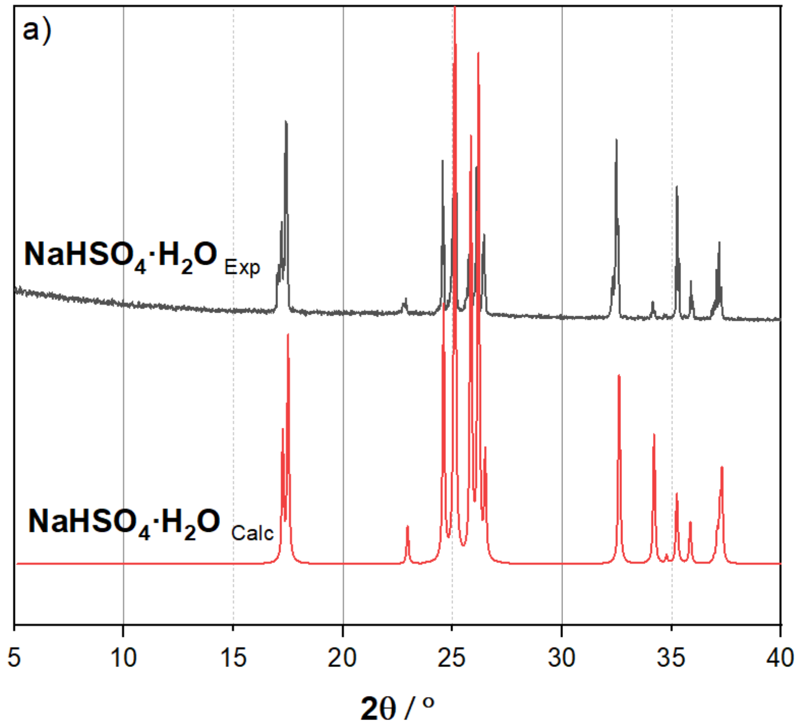

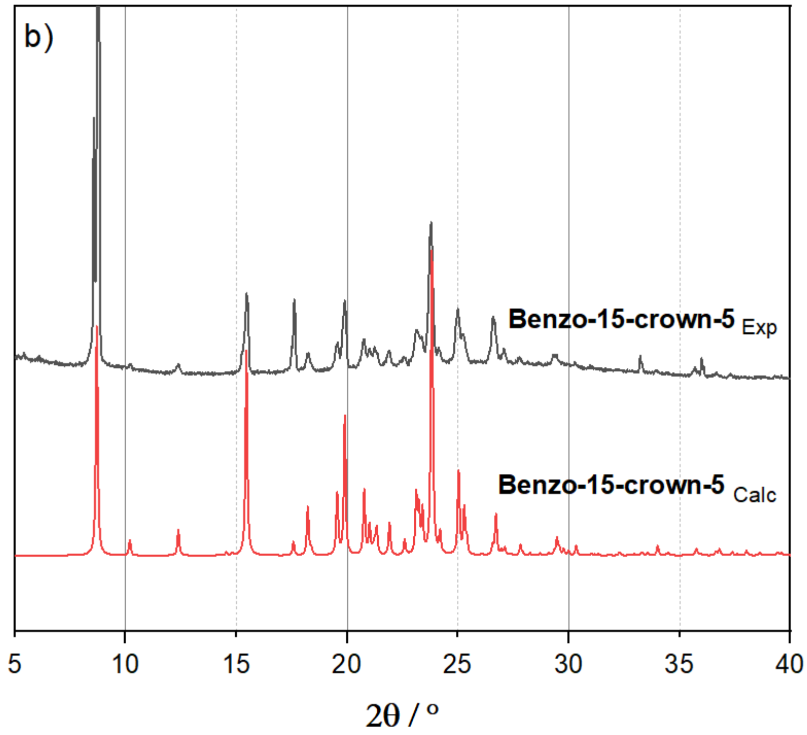

Figure SI1. Comparison between the calculated powder patterns obtained from the databases and the experimental ones of: (a) $NaHSO_4 \cdot H_2O$ (ICSD Reference code: 15066) and (b) Benzo-15-crown-5 (CCDC Reference code: OHBOCP).

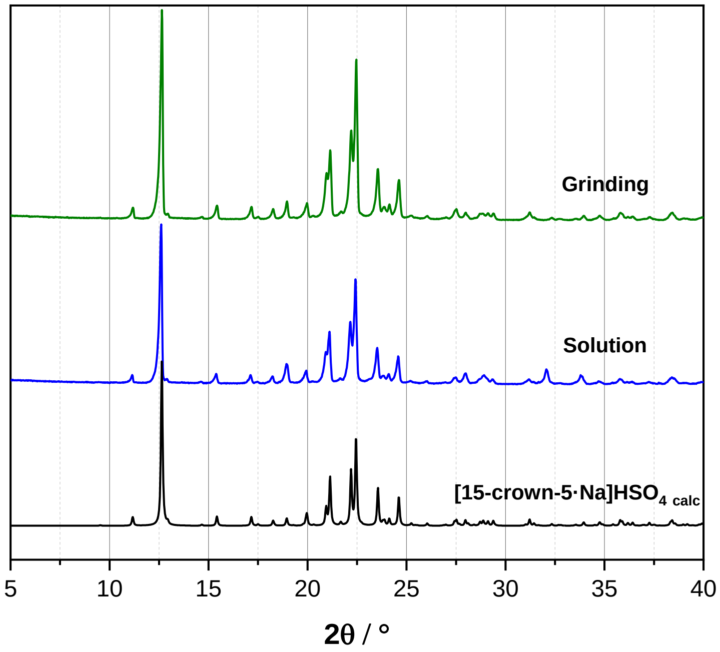

Figure SI2. Comparison between the calculated powder patterns based on the single-crystal structures of [15-crown-5·Na]HSO_4 (**1**) and the corresponding experimental ones obtained from grinding and crystallization from solution.

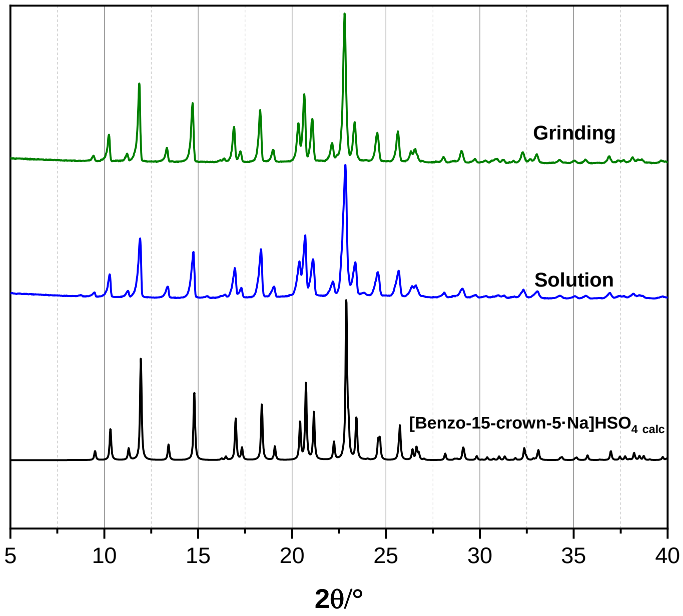

Figure SI3. Comparison between the calculated powder patterns based on the single-crystal structures of [benzo-15-crown-5·Na]HSO_4 (**2**) and the corresponding experimental ones obtained from grinding and crystallization from solution.

ATR-FTIR SPECTRA

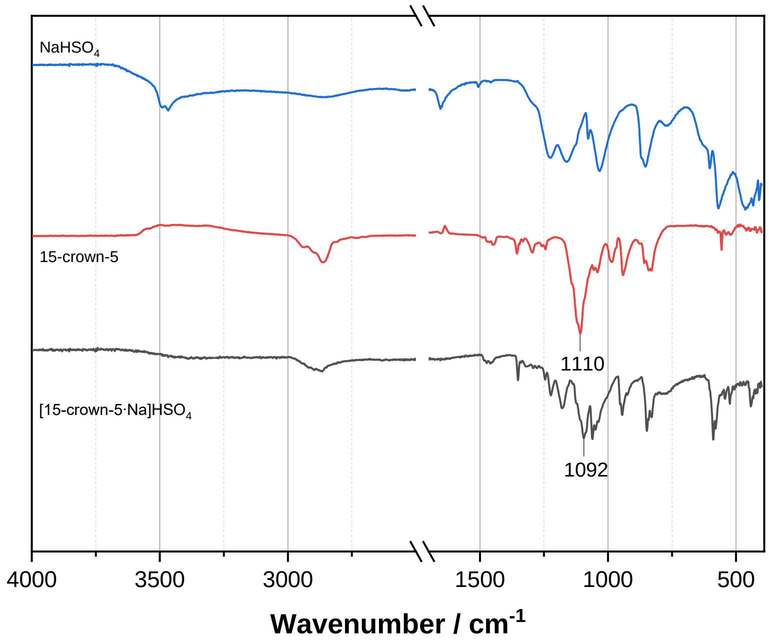

Figure SI4. Comparison between ATR-FTIR spectra recorded on polycrystalline [15-crown-5·Na]HSO_4 (**1**) and on the starting materials.

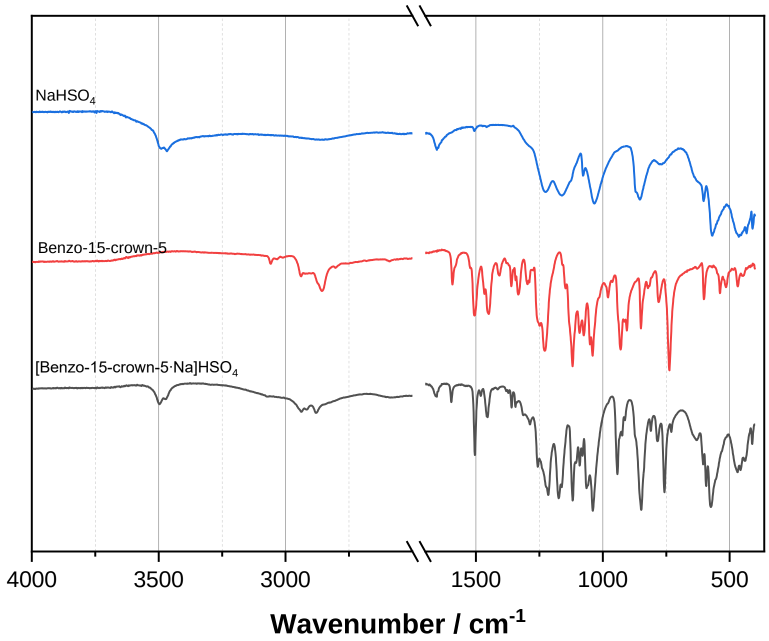

Figure SI5. Comparison between ATR-FTIR spectra recorded on polycrystalline [benzo-15-crown-5·Na]HSO_4 (**2**) and on the starting materials.

THERMOGRAVIMETRIC ANALYSES

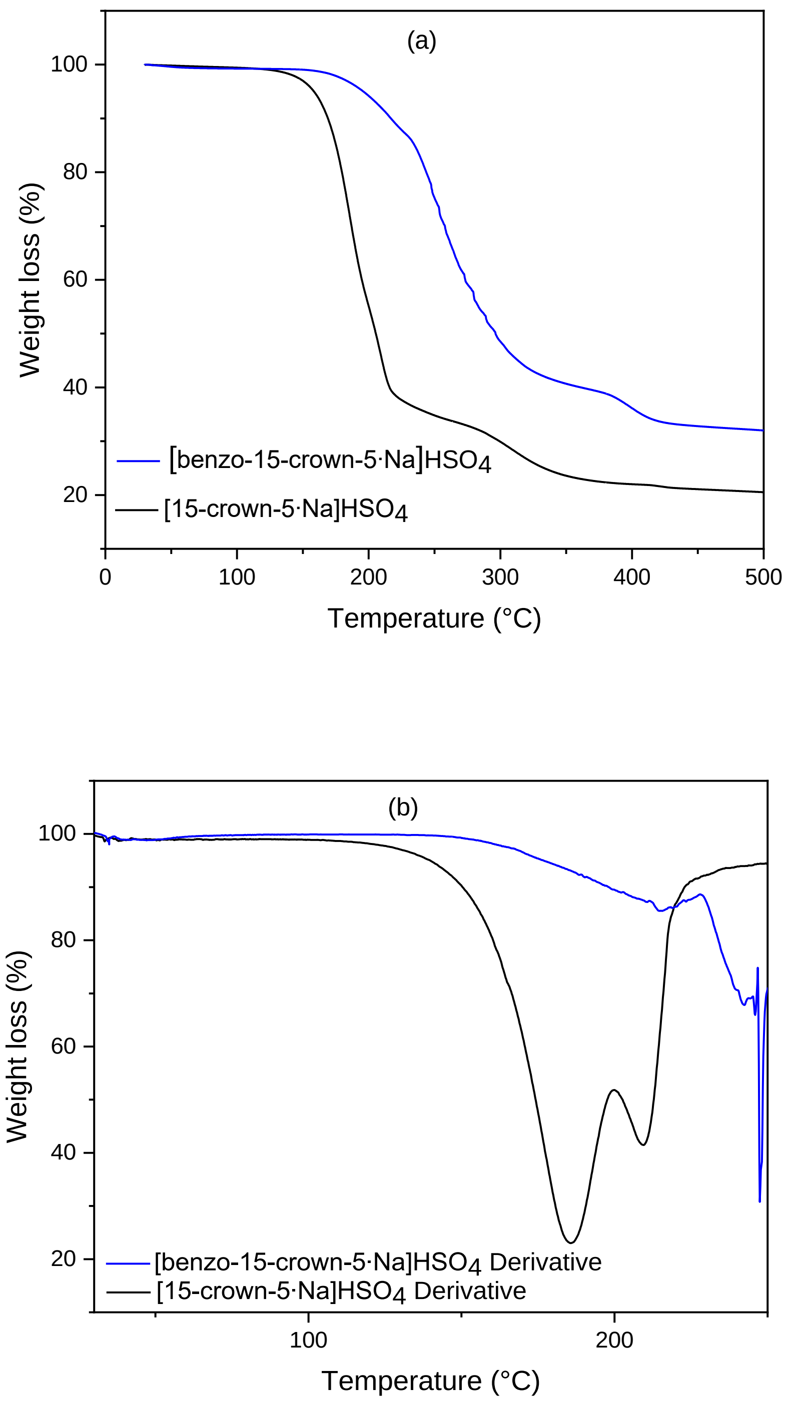

Figure SI6. (a) Thermograms recorded for the supramolecular complexes [15-crown-5·Na]HSO_4 (black line), and [benzo-15-crown-5·Na]HSO_4 (blue line). (b) The corresponding derivatives results that highlight thermal stability.

DIFFERENTIAL SCANNING CALORIMETRY TRACES

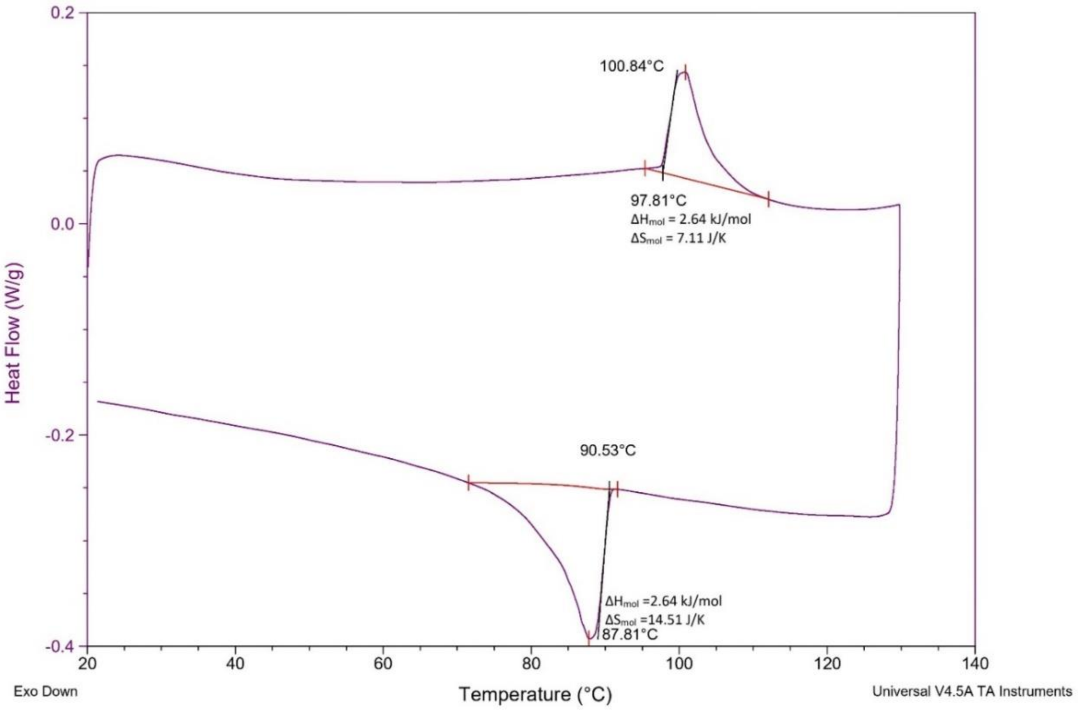

Figure SI7. DSC trace recorded on polycrystalline [15-crown-5·Na]HSO_4 (**1**).

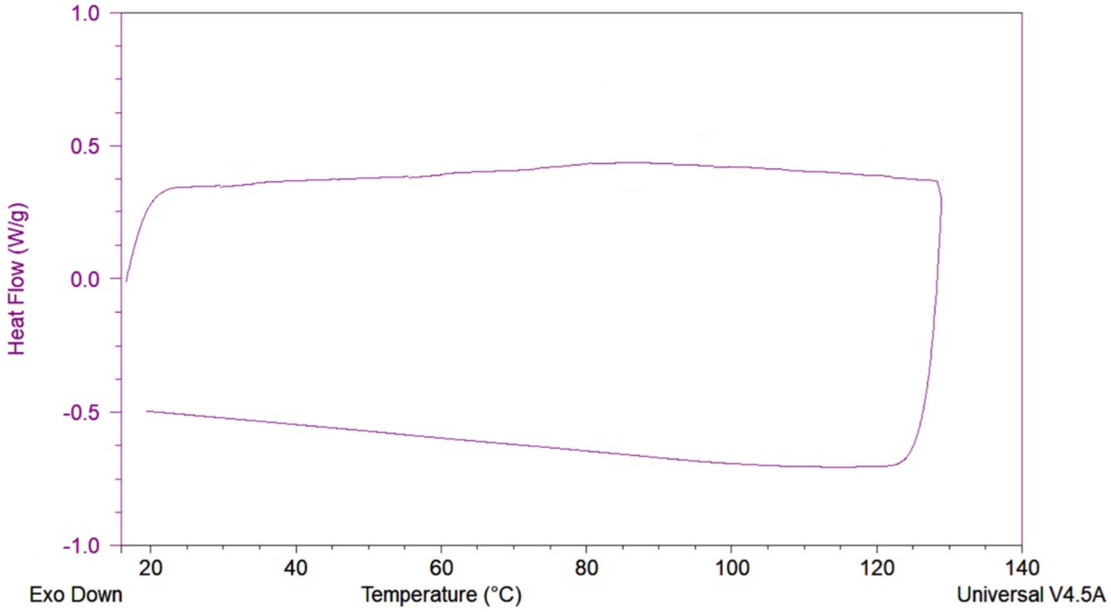

Figure SI8. DSC trace measured for polycrystalline [benzo-15-crown-5·Na]HSO_4 (**2**).

VARIABLE-TEMPERATURE PXRD PATTERNS

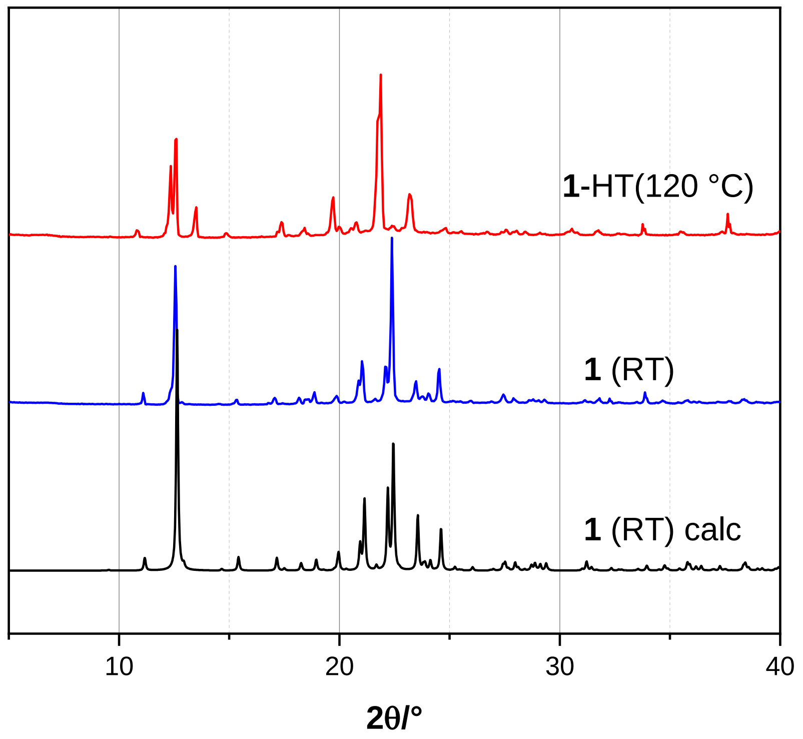

Figure SI9. VT-PXRD patterns recorded at different temperatures for polycrystalline compound **1**.

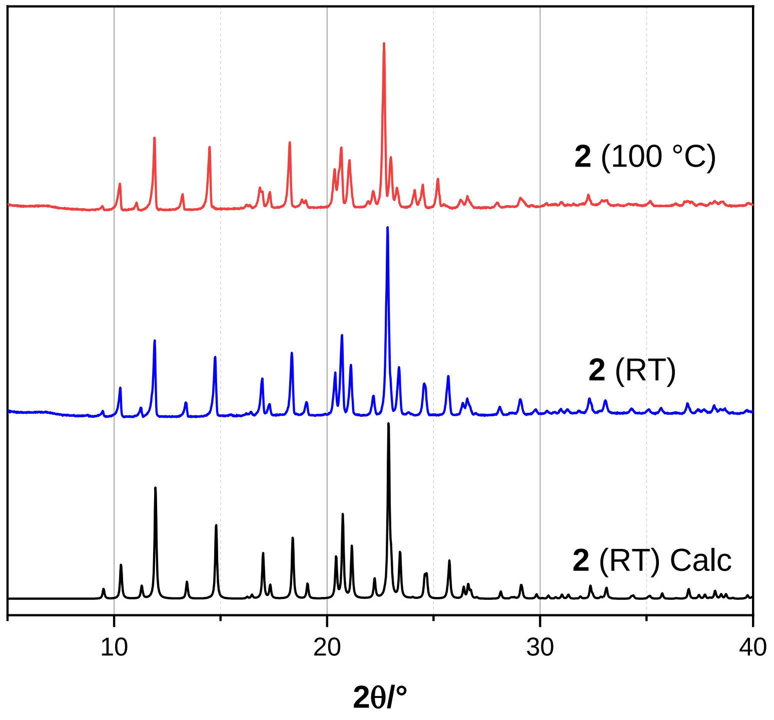

Figure SI10. VT-PXRD patterns recorded at different temperatures for polycrystalline compound **2**.

SOLID-STATE NMR SPECTROSCOPY

Table SI2. List of set temperatures (in °C) employed for the ^{1}H T_1 SSNMR study, with the corresponding sample T values (both in °C and in K), according to the temperature calibration procedure with $Pb(NO_3)_2$ (see Experimental Section).

Set T (°C)	Sample T (°C)	Sample T (K)
20.0	31.3	304.4
25.0	35.7	308.9
30.0	40.2	313.4
40.0	49.1	322.3
50.0	58.1	331.3
60.0	67.0	340.2
65.0	71.5	344.7
70.0	76.0	349.2
75.0	80.4	353.6
80.0	84.9	358.1
85.0	89.4	362.6
90.0	93.8	367.0
95.0	98.3	371.5
100.0	102.8	376.0
105.0	107.2	380.4
110.0	111.7	384.9
115.0	116.2	389.4
120.0	120.7	393.9

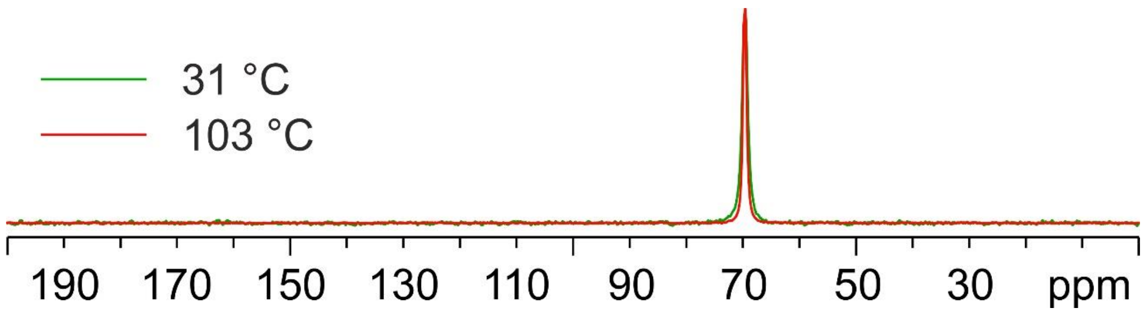

Figure SI11. ^{13}C (150.91 MHz) CPMAS SSNMR spectra of **1**, acquired at the indicated temperatures at a spinning speed of 12 kHz.

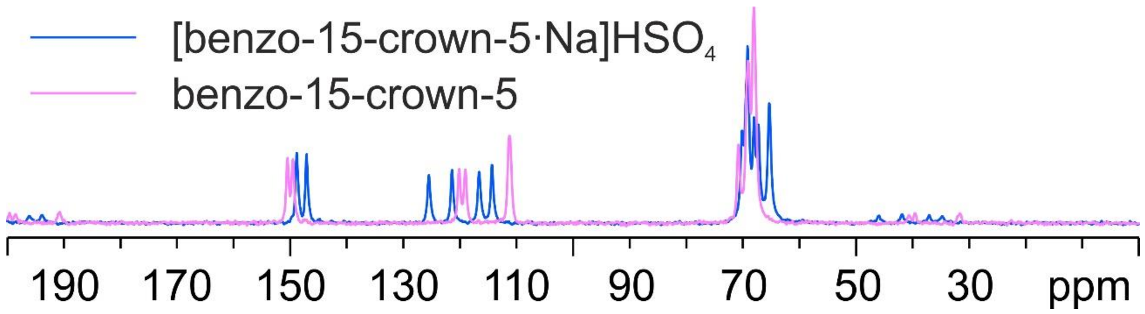

Figure SI12. ^{13}C (150.91 MHz) CPMAS SSNMR spectra of **2** (in blue) and pure benzo-15-crown-5 (in pink), acquired at probe temperature at a spinning speed of 12 kHz.

Table SI3. ^{13}C SSNMR chemical shifts with assignment for [15-crown-5·Na]HSO_4, [benzo-15-crown-5·Na]HSO_4 and benzo-15-crown-5.

[15-crown-5·Na]HSO_4		[benzo-15-crown-5·Na]HSO_4		benzo-15-crown-5	
^{13}C chemical shift (ppm)	Assignment	^{13}C chemical shift (ppm)	Assignment	^{13}C chemical shift (ppm)	Assignment
69.6	**C**H_2	148.8	Aromatic **C**$_q$	150.4	Aromatic **C**$_q$
		147.0	Aromatic **C**$_q$	149.5	Aromatic **C**$_q$
		125.4	Aromatic **C**H	120.1	Aromatic **C**H
		121.3	Aromatic **C**H	119.0	Aromatic **C**H
		116.5	Aromatic **C**H	111.2	2 x Aromatic **C**H
		114.3	Aromatic **C**H	70.7	**C**H_2
		70.0	**C**H_2	69.0	3 x **C**H_2
		69.4	**C**H_2	68.0	4 x **C**H_2
		69.1	2 x **C**H_2		
		68.0	**C**H_2		
		67.2	**C**H_2		
		65.3	2 x **C**H_2		

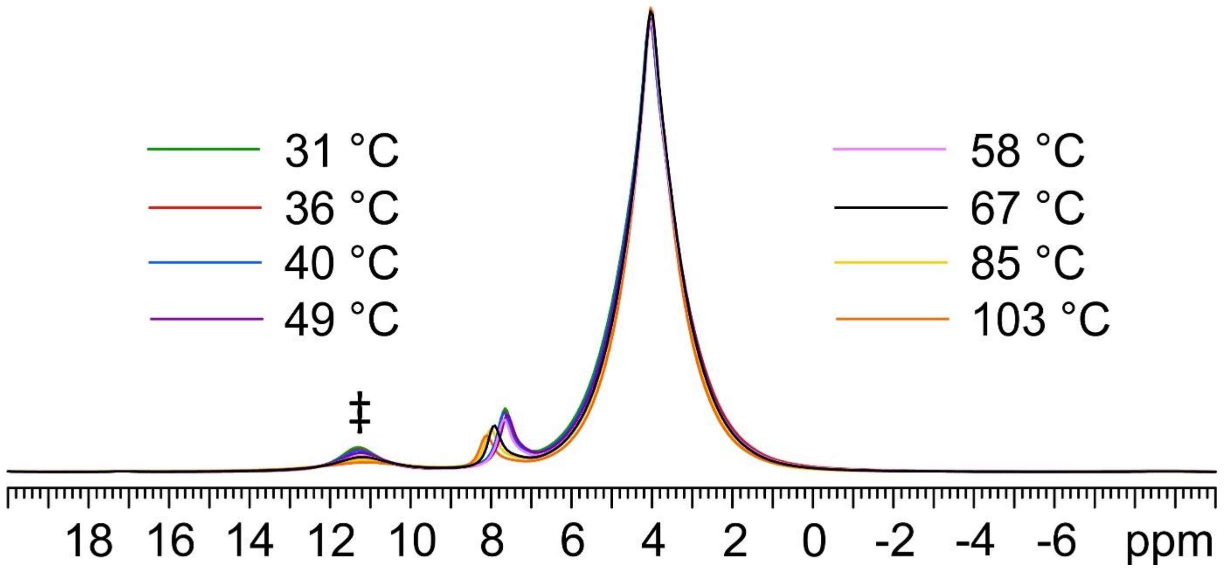

Figure SI13. ^{1}H (600.13 MHz) MAS SSNMR spectra of **1**, acquired at the indicated temperatures at a spinning speed of 12 kHz. The symbol "‡" indicates an unassignable signal.

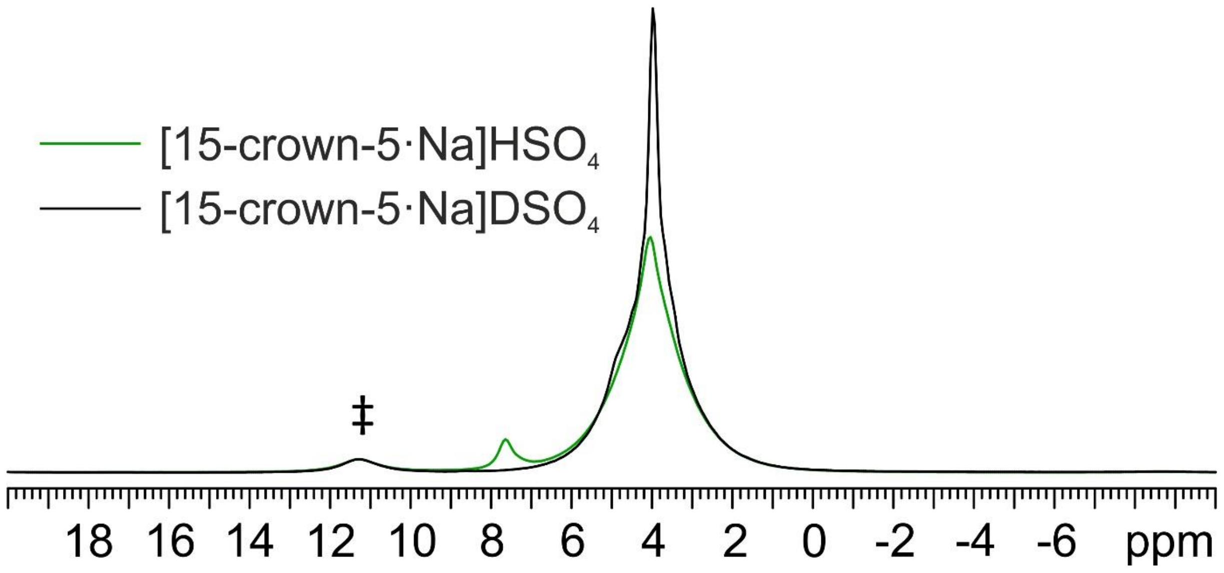

Figure SI14. ^{1}H (600.13 MHz) MAS SSNMR spectra of **1** (in green) and its $NaDSO_4$-based analogous (in black), acquired at probe temperature at a spinning speed of 12 kHz. The symbol "‡" indicates an unassignable signal.

ELECTROCHEMICAL IMPEDANCE SPECTROSCOPY (NYQUIST PLOTS)

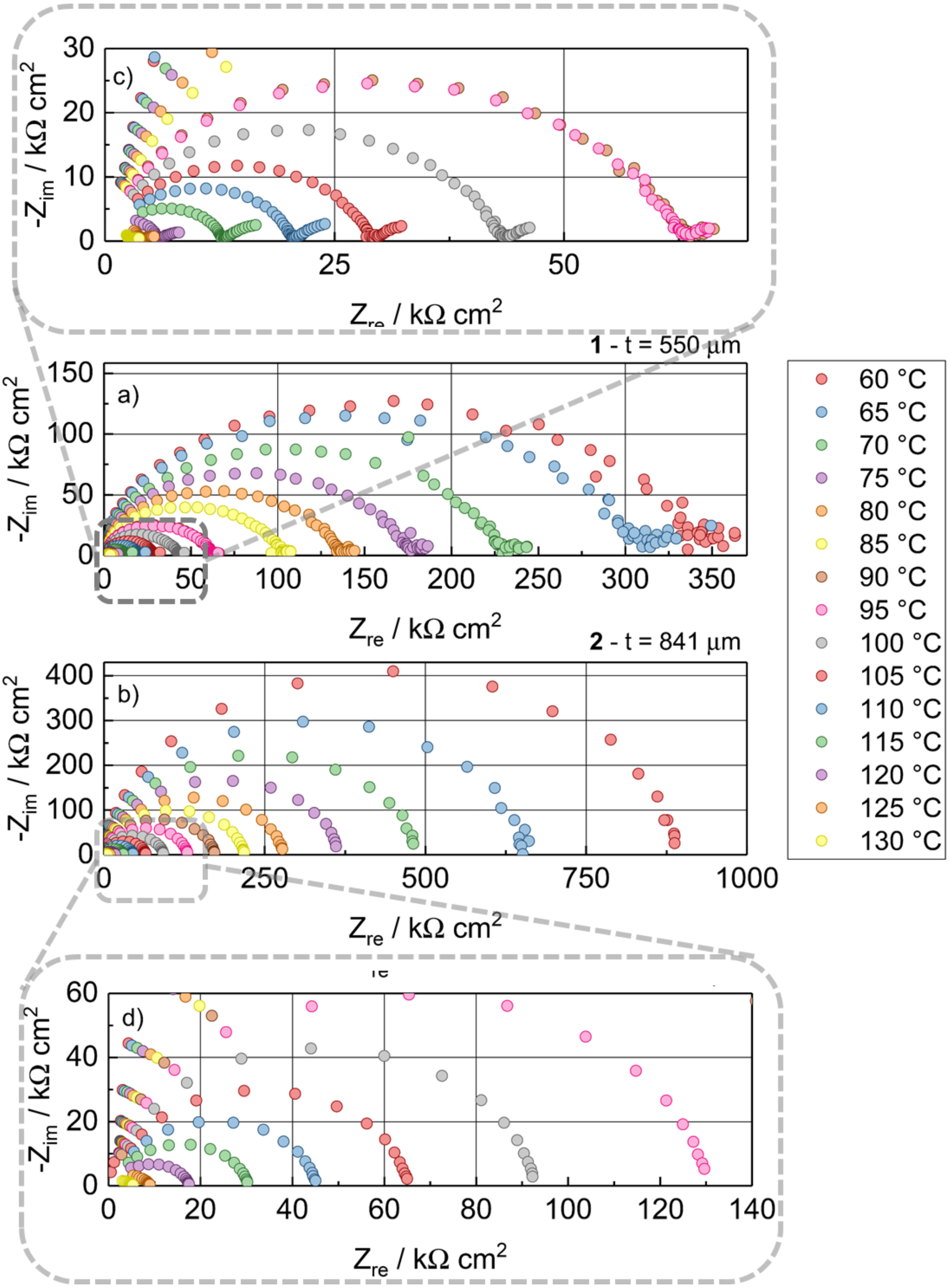

Figure SI15. Nyquist plots at different temperatures of: (a) [15-crown-5·Na]HSO_4 (**1**) and (b) [benzo-15-crown-5·Na]HSO_4 (**2**).
